# Safety and feasibility of early discharge (≤ 23 h) following loop ileostomy reversal: a systematic review and meta-analysis

**DOI:** 10.1007/s00384-026-05150-6

**Published:** 2026-05-30

**Authors:** Cormac B. Mulhall, Ronan O’Kelly, Hugo C. Temperley, Benjamin M. Mac Curtain, Michelle Dunne, Zi Qin Ng

**Affiliations:** 1 Department of Health Sciences, University of Galway, Galway, Ireland; 2 Dept of General Surgery, Royal Perth Hospital, Perth, WA 6000 Australia; 3 Department of Radiology, St James’s Hospital, Dublin, D08 NHY1 Ireland; 4 Trinity College Dublin, St James’s Cancer Institute, Dublin, D08 NHY1 Ireland; 5 Department of Urology, Northwell Health, New Hyde Park, NY USA; 6 Department of Urology, Lenox Hill Hospital, New York, NY 10075 USA

**Keywords:** Stoma reversal, Colorectal surgery, Enhanced recovery after surgery

## Abstract

**Background:**

The standard practice for postoperative management of loop ileostomy reversal has been standard admission with several days of inpatient observation, largely to monitor for postoperative complications. These primarily include anastomotic leak, postoperative ileus, and surgical site infection. In recent years, several studies have piloted ambulatory or short-stay pathways; however, the safety and impact of these pathways on complications remains unclear.

**Methods:**

This systematic review and meta-analysis was conducted in accordance with PRISMA. It was also conducted in accordance with a prospectively registered protocol (PROSPERO CRD420251252408). Adults undergoing loop ileostomy reversal discharged within 23 h of surgery (including same-day discharge (SDD), short-stay protocols (LOS 1)) were compared with patients managed with standard admission. Primary outcomes were anastomotic leak, postoperative ileus, and surgical site infection at 30 postoperative days; secondary outcomes included readmission, overall complications including Clavien-Dindo-graded events, and mortality. Random-effects models were used to pool odds ratios with 95% confidence intervals.

**Results:**

Twelve studies (11 retrospective cohort studies, 1 RCT), comprising 30,040 patients were included. Of these, 2611 (8.7%) patients underwent ambulatory or short-stay reversal. There was no significant difference between early discharge and standard cohorts in anastomotic leak (pooled OR 1.31, 95% CI 0.24–7.32), postoperative ileus (pooled OR 0.49, 95% CI 0.16–1.55), or surgical site infection (pooled OR 0.76, 95% CI 0.38–1.51). However, the low event rates and wide Cis likely preclude confident exclusion of a clinically meaningful effect. Similarly, readmission rates showed no difference between groups (pooled OR 0.97, 95% CI 0.78–1.19). Early discharge following reversal was, however, associated with a modest but statistically significant reduction in overall postoperative complications (pooled OR 0.70, 95% CI 0.50–0.98), with comparable rates of major (Clavien-Dindo III–IV) complications and very low mortality in both groups.

**Conclusions:**

This study suggests that early discharge following loop ileostomy reversal, if carried out as part of a structured perioperative pathway, appears to be safe, with no apparent increase in risk of any additional postoperative complications. However, the data available at present is dominated by largely retrospective cohort studies, with heterogenous discharge criteria, and inconsistency in intervention (SDD vs 23 h stay vs short-stay protocol), precluding any firm conclusions regarding equivalence with standard admission. Rather than advocating for its widespread adoption, these findings support the cautious implementation of early discharge pathways in select cohorts with prospectively collected outcomes. Adequately powered multicentre RCTs with standardisation of intervention and outcome measures are required before broader dissemination can be recommended.

**Clinical trial registration:**

Not applicable. This study is not a clinical trial.

**Supplementary Information:**

The online version contains supplementary material available at 10.1007/s00384-026-05150-6.

## Introduction

Defunctioning loop ileostomy formation is a crucial component of colorectal surgery, particularly in the management of low rectal cancer. In the UK, there are an estimated 20,000 stomas formed annually, with 80% of these being formed in the elective setting [1]. In Australia, there are an estimated 8600 new stomas formed each year, with a high percentage of these being reversed at 6 months [2]. This procedure accounts for a high number of bed days and high costs. Since 2003, there has been a slow-growing shift towards early discharge of this cohort [3], augmented by the advent of ERAS principles [4]. With the implementation of these principles, postoperative care in colorectal surgery has become more efficient, leading to decreased length of stay [5]. Despite this, many stoma reversal patients are kept as inpatients pending return of normal gut function, despite no evidence of this being indicated or beneficial [6–8]. The emergence of early discharge pathways in this cohort emanates from Kalady et al., who in 2003 achieved safe, 23-h discharge in 28 carefully selected patients [3]. This inspired several other pilot studies to assess the clinical safety and feasibility of short-stay reversal (SS) and same-day discharge reversal (SDD).

Loop ileostomy reversal is generally considered a safe procedure associated with a mortality of 0.4–2.4% [10, 11]. Reported morbidity risk in the literature is 17–22% [11, 12] with main complications postoperatively including ileus (2–6.4%), anastomotic leak (< 1%), and wound infection (1.5–3%) [12, 13]. Importantly, these complications tend to declare themselves several days postoperatively, potentially mitigating the need for prolonged inpatient observation.

Despite these promising results, there has been a lag to introduce early discharge pathways for this cohort. In this review, we aim to accumulate and systematically analyse the safety and feasibility of same-day discharge with regard to complication rate. By synthesising these data, we hope to identify key factors favourable for same-day discharge.

## Methods

### Protocol and registration

This systematic review was conducted in accordance with the Preferred Reporting Items for Systematic Review and Meta-analysis (PRISMA) statement [14]. Prior registration published on PROSPERO on 12th December 2025 (registration number CRD420251252408).

### Eligibility criteria

Study eligibility was defined according to a drafted PICO framework.

#### Population

Adults undergoing loop ileostomy reversal were included.

#### Intervention

Our intervention group included patients who were discharged from the hospital within 23 h of their procedure being completed, including SDD, short-stay protocols where LOS < 1, and 23-h stay pathways.

#### Comparator

The intervention group was compared to patients having a standard admission, i.e. patients who were not on an established early discharge pathway.

#### Outcomes

Primary outcomes were anastomotic leak, surgical site infection, and postoperative ileus on the 30th postoperative day. Secondary outcomes included need for readmission, total complication rate and mortality.

Primary outcomes:

i. Anastomotic leak: defined as “a defect of the intestinal wall integrity at the anastomotic site leading to a communication between the intra- and extraluminal compartments”, as per the International Study Group of Rectal Cancer (ISREC) definition [15, 16].
ii. Postoperative ileus was defined as “a temporary inhibition of coordinated propulsive bowel motility following surgery, characterised by two or more of the following occurring on or after postoperative day 4: nausea or vomiting, inability to tolerate an oral diet over 24 h, absence of passage of flatus over 24 h, abdominal distension, or radiological confirmation of ileus”, as per the consensus definition proposed by Vather et al. [17].
iii. Surgical site infection was defined as “infection occurring within 30 days of colorectal surgery” and was categorised as superficial, deep, or organ/space, consistent with definitions used in large colorectal cancer SSI cohorts and reviews [18–20]. Where studies did not subclassify SSI, only the overall incidence of SSI was extracted and analysed.

Secondary outcomes:

i. Readmission was defined as any unplanned return to the hospital within 30 days of reversal, irrespective of the cause for admission.
ii. Total complications were defined as any unplanned deviation from the normal postoperative course, graded according to the Clavien-Dindo classification system, where available [21].

### Eligibility criteria

Inclusion criteria:

1. Adult patients over 18 years of age undergoing reversal of loop ileostomy
2. Patient discharged within 23 h of intervention
3. Published in the English language

Exclusion criteria:

1. Children under 18 years of age
2. Patients undergoing other types of stoma reversal or concomitant procedures
3. Consensus opinions, case series with less than 10 patients, case reports
4. Studies failing to capture at least one of three primary outcomes

### Information sources and search strategy

Complete literature searches were conducted in PubMed and Scopus from inception to 16 December 2025. No language restrictions were used. Combined terms related to stoma reversal, same-day discharge, early discharge protocols, and complications, including “Stoma Reversal”, “Early discharge”, “Same-day discharge”, “Loop ileostomy”, “Ambulatory”, “23-hour discharge”, “Anastomotic Leak”, “Surgical Site Infection”, and “Postoperative Ileus”, were included. Reference lists of all included studies and relevant papers were screened. Furthermore, grey literature sources were searched for additional eligible studies. Full search strategy can be found in supplementary materials.

### Study selection

Studies from above search were imported into review management software Covidence. All duplicates were removed. Titles and abstracts were screened independently by two reviewers (CM and RO’K). Next, eligible studies underwent full-text review, comparing against pre-determined inclusion criteria and ineligible studies excluded. Where there were disagreements between reviewers, disputes were resolved by consensus or adjudication by a third reviewer (ZN).

### Data extraction

Data was extracted using a pre-piloted form using Covidence. Data relating to study characteristics (year, study type, and country), patient demographics (age, BMI, ASA, comorbidities, type of follow-up, etc.), and outcomes (leak, ileus, infection, readmission, morbidity, and mortality). Discrepancies were resolved by a third reviewer/adjudicator (ZN).

### Data synthesis

Statistical analyses were performed using Stata version 18 (StataCorp. 2021. *Stata Statistical Software: Release 18*. College Station, TX: StataCorp LLC). Log-ORs were pooled using a random-effects model with restricted maximum likelihood estimation with 95% confidence intervals. Pooled log ORs are displayed as exponentials of the log for clarity. Heterogeneity among studies was assessed using the Tau^2^, *H*^2^, and *I*^2^ statistics. An *I*^2^ value greater than 50% was interpreted as evidence of substantial heterogeneity. Due to expected variability in study design and populations, a random-effects model was employed. Evidence was assessed using GRADE approach, tabulated results of which can be found in supplementary materials. A pre-specified sensitivity analysis was undertaken excluding the three large retrospective database studies [31–33], which together contributed the majority of the pooled sample but were considered most susceptible to selection bias, per peer review suggestion.

### Risk of bias

Two tools were used for calculating risk of bias of included studies. Potential biases within the included RCT were assessed using the Cochrane Collaboration tool [22], with the Newcastle- Ottawa scale (NOS) risk of bias tool [23] being used for non-RCT studies. Cochrane RoB grades each study as being high, low, or unclear risk of bias across six categories. NOS tool grades each study as being “satisfactory” or “unsatisfactory” across a number of parameters. The critical appraisal was completed by two reviewers independently (CM and ROK). A third reviewer was asked to arbitrate in the event of discrepancy of opinion (ZN).

The applied methodological approach was adapted from our group’s previous studies [24–26], with modifications made to account for this specific study’s objectives and available dataset.

## Results

### Study selection and characteristics

Our systematic search identified [6, 9, 12, 27–36] 12 studies meeting predefined inclusion criteria, comprised of 11 observational studies and one randomised control trial. Studies ranged from 2015 to 2025. Some studies were pilot studies in trialling same-day discharge pathways with dedicated follow-up, while some studies retrospectively examined outcomes in patients discharged early without having a specific protocol in place. Only one large ACS-NSQIP analysis reported indication for ileostomy reversal in a malignant/benign framework. Across 6160 reversals, 395 (6.4%) were coded as malignant and 522 (8.5%) as benign, with the remainder classified as “unknown”. Within that cohort, early discharge (POD 0–1) and standard discharge (POD 2–3) groups had similar distributions of malignant (4.5% vs 6.5%) and benign (8.7% vs 8.4%) indications, in keeping with reported proportions of other studies included [27–30, 32]. Pilot studies typically excluded patients with ASA Grade > III, significant cardiorespiratory comorbidity, inadequate social support, or those undergoing concomitant procedures [6, 9, 27, 29, 32, 35], whereas larger registry-based analyses and more recent studies applied no prospective exclusion criteria, with discharged determined post hoc or at surgeon discretion [28, 30, 31, 33, 34, 36].

### Risk of bias

The included RCT [27] was calculated to be low risk of bias, using the Cochrane Collaboration risk of bias assessment for RCTs. For included non-RCT studies, one study was “very good”, eight studies were “good”, two studies were “satisfactory”, and zero studies were “unsatisfactory”. A summary of this risk of bias assessment is included in supplementary materials.

### Patient demographics

Thirty thousand and forty patients were included for analysis. Of those patients, mean BMI was 26.3 kg/m^2^, with the mean age being 57.6 years. There was considerable discrepancy in reporting of American Society Anaesthesiologist Grade between studies [37]. Four hundred sixty patients were assigned an allocated grade, of which the majority were ASA Grade II (383) [6]. Another study classified patients as less than grade III (122) and grade III or more (101) [31]. Where reported, anastomotic reconstruction was predominantly stapled side-to-side, accounting for 252 of 325 closures (77.5%), with 73 of 325 (22.5%) performed using hand-sewn techniques [9, 27, 32]. Where reported, purse-string skin closure was almost universal (75/77 procedures across two studies), but most other series did not describe skin closure in sufficient detail to permit pooling [24, 27]. Patient demographic data can be found illustrated in Table 1.

**Table 1:** Demographic data

Author	Year	Country	Sample size	Mean age	Mean BMI	Type of stoma	Discharge criteria	Type of closure
Peacock	2011	UK	23	63	-	Loop ileostomy	+	Staples
Bhalla	2015	UK	15	67	-	Loop ileostomy	+	Staples
Azin	2017	Canada	6160	50.03	26.4	Loop ileostomy	-	-
Taylor	2019	USA	668	-	-	Loop ileostomy	-	-
Nguyen	2020	Canada	117	61	25.5	Loop ileostomy	-	Staples
Allart	2021	France	236	54	24.7	Loop ileostomy	-	Both
Afshari	2022	Sweden	60	67	27	Loop ileostomy	+	-
Shen	2023	China	236	55	23.5	Loop ileostomy	-	Staples
Charbonneau	2024	Canada	47	59.5	27.5	Loop ileostomy	+	Both
Ferrari	2024	USA	64	50	27.4	Loop ileostomy	+	Both
Ferrari	2025	USA	22,312	53.5	28.1	Loop ileostomy	-	-
Wang	2025	Canada	102	57.6	26.3	Loop ileostomy	-	-

### Discharge criteria

Discharge criteria varied considerably between studies. Five studies specified criteria which needed to be met for the patient to be discharged successfully as same-day discharge [9, 27, 31, 32]. These criteria included postoperative tolerance of diet, ambulating independently and return of bladder and bowel function in some cases. The remaining included studies either did not refer to discharge criteria or discharge postoperatively was at the discretion of the operating surgeon [29, 30, 32–35, 37].

### Primary outcomes

#### Anastomotic leak (Fig. 1)

**Fig. 1:**
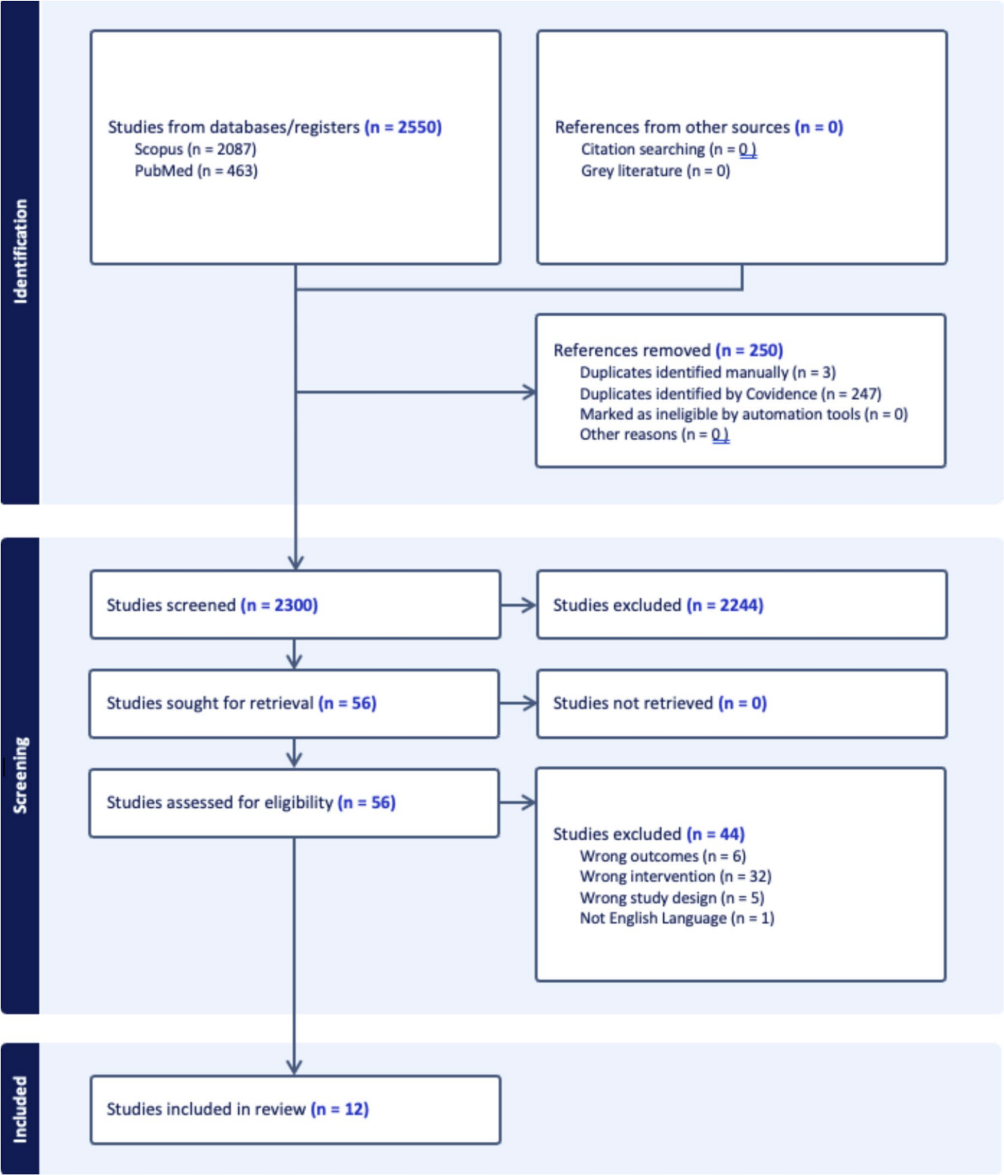
PRISMA flow diagram summarising study selection

Four studies contributed data on anastomotic leak [29, 30, 34, 36] (Table 2). Between-study heterogeneity was moderate (*I*^2^ = 33.2%, *p* = 0.03). There was no clear effect of early discharge pathways compared with standard admission (pooled odds ratio [OR] 1.31, 95% confidence interval [CI] 0.24–7.32).

**Table 2:** Overall complications by Clavien-Dindo classification

Study	Group	*n* patients	Any complication, *n* (%)	Grades I–II, *n* (%)	Grades III–IV, *n* (%)	Grade V, *n* (%)
Allart	Day-case PP cohort	40	12 (30.0)	9 (22.5)	3 (7.5)	0 (0)
	Inpatient (non-DCS)	186	62 (33.3)	45 (24.2)	16 (8.6)	1 (0.5)
Charbonneau	23-h stay ERAS	24	5 (20.8)	4 (16.7)	1 (4.2)	0 (0)
	Conventional admission	23	5 (21.7)	5 (21.7)	0 (0)	0 (0)
Afshari	23-h stay protocol	30	7 (23.3)	5 (16.7)	2 (6.7)	0 (0)
	Historical admitted cohort	30	7 (23.3)	5 (16.7)	2 (6.7)	0 (0)
Taylor	Outpatient < 23 h	471	45 (9.6)	34 (7.2)	11 (2.3)	0 (0)
	Inpatient > 23 h	768	133 (17.3)	98 (12.8)	35 (4.6)	0 (0)
Wang	ASC same-day discharge	12	5 (41.7)	3 (25.0)	2 (16.7)	0 (0)
	Hospital SDD	64	15 (23.4)	11 (17.2)	4 (6.3)	0 (0)
Ferrari	SDD (LOS 0)	201	14 (7.0)	8 (4.0)	5 (2.5)	1 (0.5)
	Short stay (LOS 1)	1833	110 (6.0)	81 (4.4)	25 (1.4)	4 (0.2)
	Inpatient (LOS ≥ 2)	20,278	2,395 (11.8)	1,879 (9.3)	404 (2.0)	112 (0.6)

#### Postoperative ileus (Figs. 2 and 3)

**Fig. 2:**
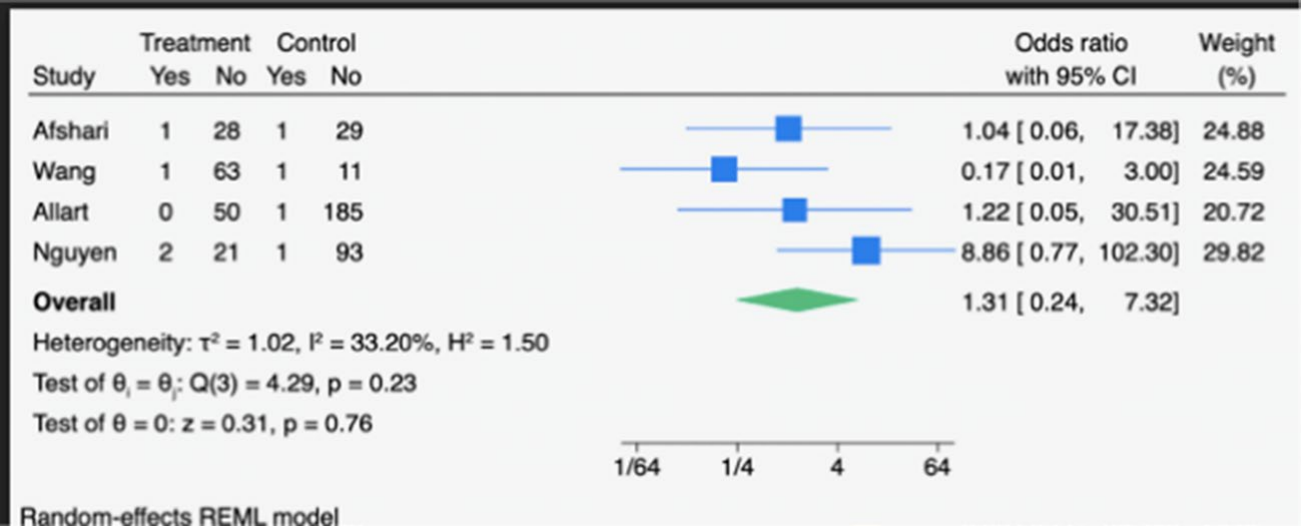
Forest plot of anastomotic leak comparing ambulatory or short-stay stoma reversal with standard inpatient admission

**Fig. 3:**
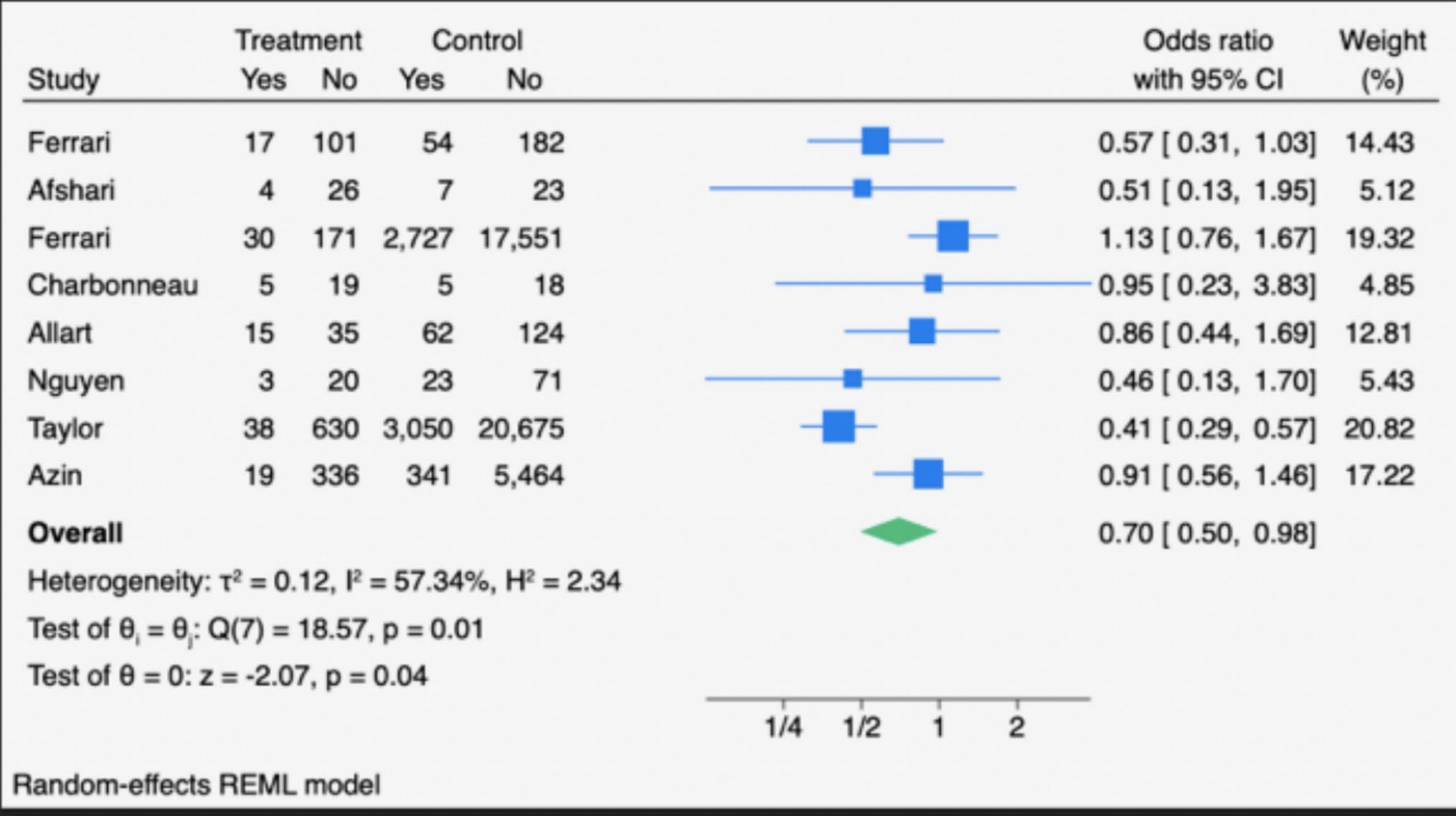
Forest plot of overall postoperative complications comparing ambulatory or short-stay and standard inpatient stoma reversal pathways

Five studies provided data on postoperative ileus [27, 30, 32, 33, 34 s ^27,30,32,33.34,36^. Heterogeneity was substantial (*I*^2^ = 65.4%, *p* = 0.03). The pooled effect favoured early discharge management but was not statistically significant (pooled OR 0.49, 95% CI 0.16–1.55).

#### Surgical site infection (Fig. 4)

**Fig. 4:**
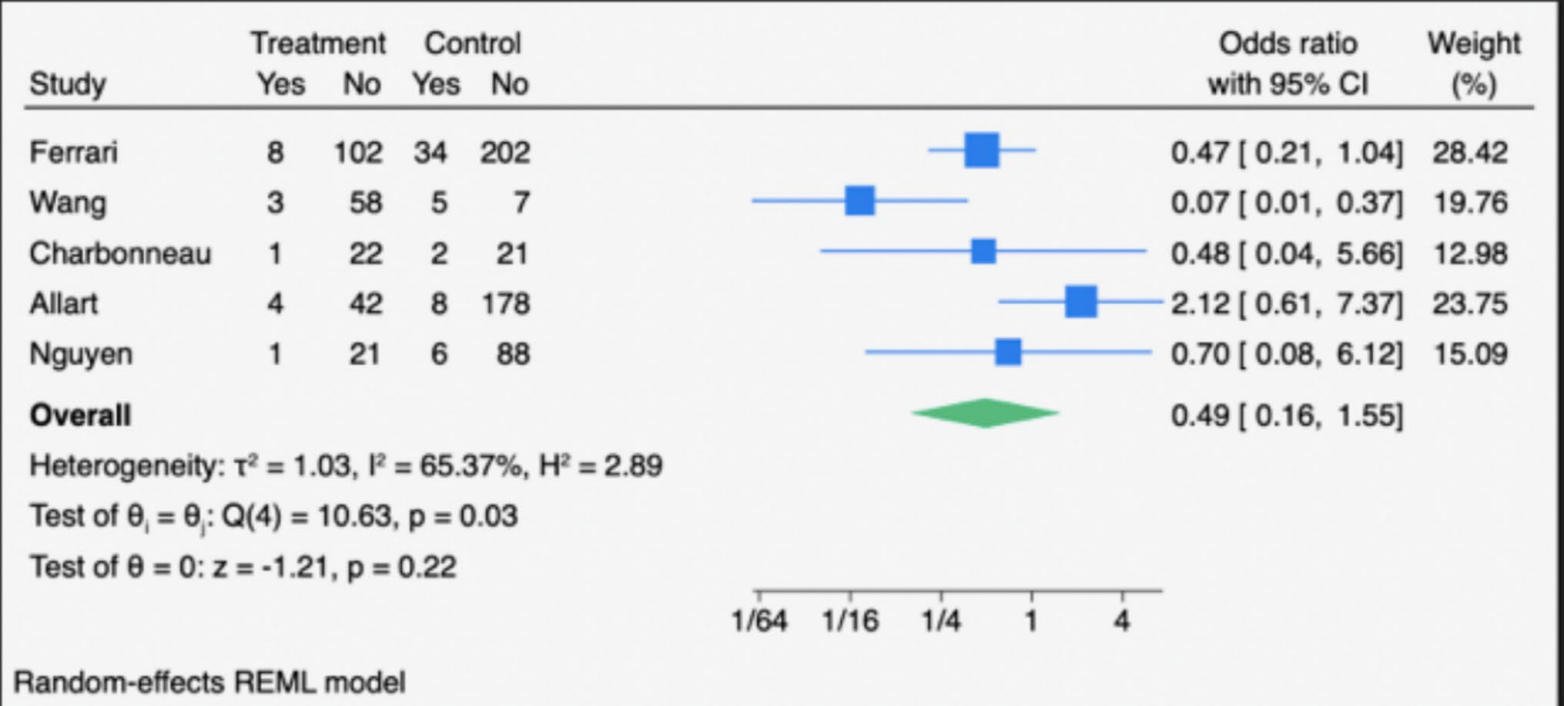
Forest plot of postoperative ileus rates for ambulatory or short-stay versus standard inpatient stoma reversal

Of 12 included studies, six were suitable for analysis of SSI [6, 27, 29, 30, 32, 36]. Heterogeneity was low (*I*^2^ = 23.7%, *p* = 0.26). We found no significant difference in surgical site infection rates between early discharge and standard pathways (pooled OR 0.76, 95% CI 0.38–1.51).

### Secondary outcomes

#### Readmission (Fig. 5)

**Fig. 5:**
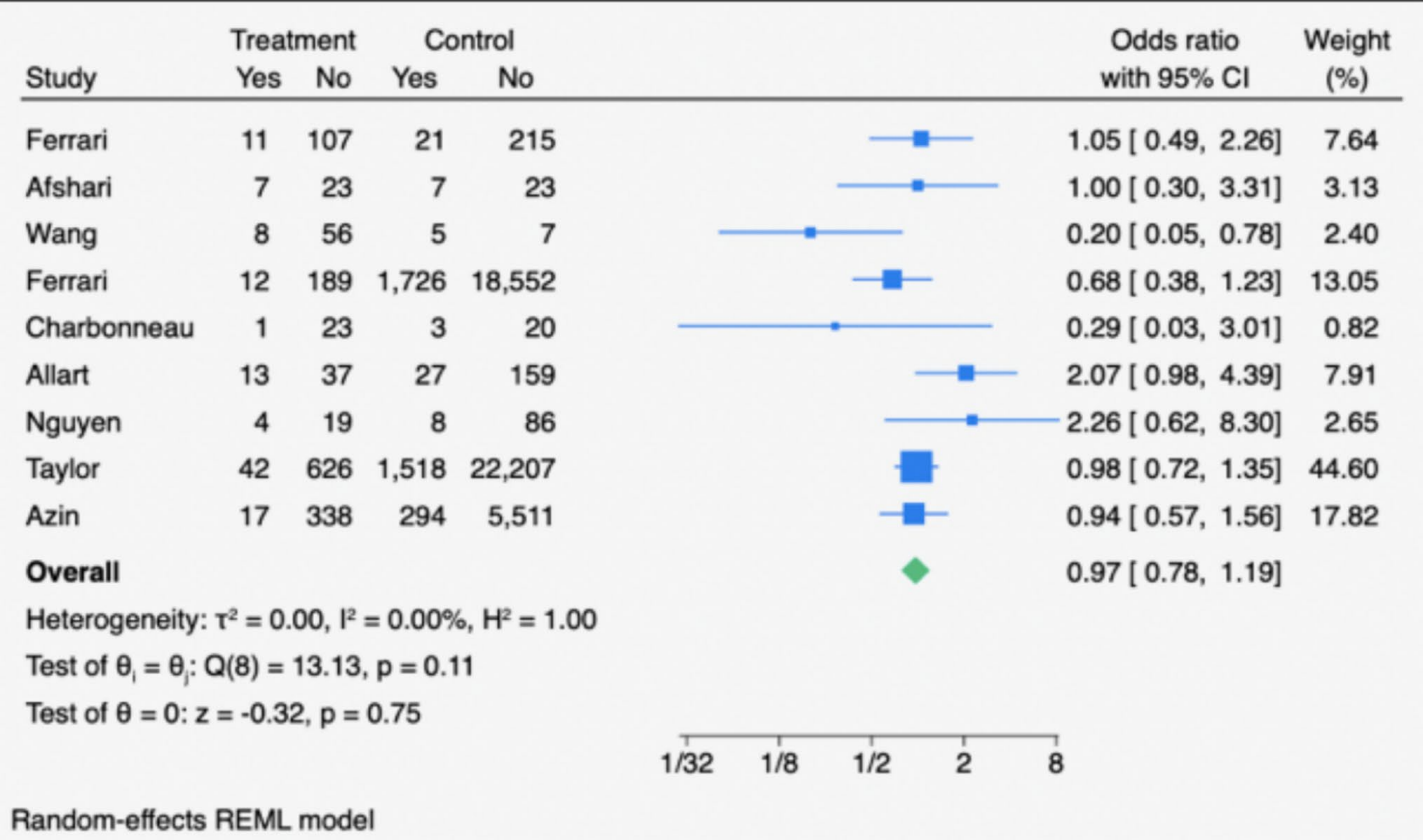
Forest plot of unplanned 30-day hospital readmission following ambulatory or short-stay versus standard stoma reversal

Nine studies reported hospital readmission [9, 27, 29–31, 33, 36–38]. No evidence of between-study heterogeneity was found (*p* = 0.11). There was no evidence of between-study heterogeneity (*p* = 0.11). Pooled analysis showed no significant difference in readmission between early discharge and standard care (pooled OR 0.97, 95% CI 0.78–1.19).

#### Overall complications (Fig. 6)

**Fig. 6:**
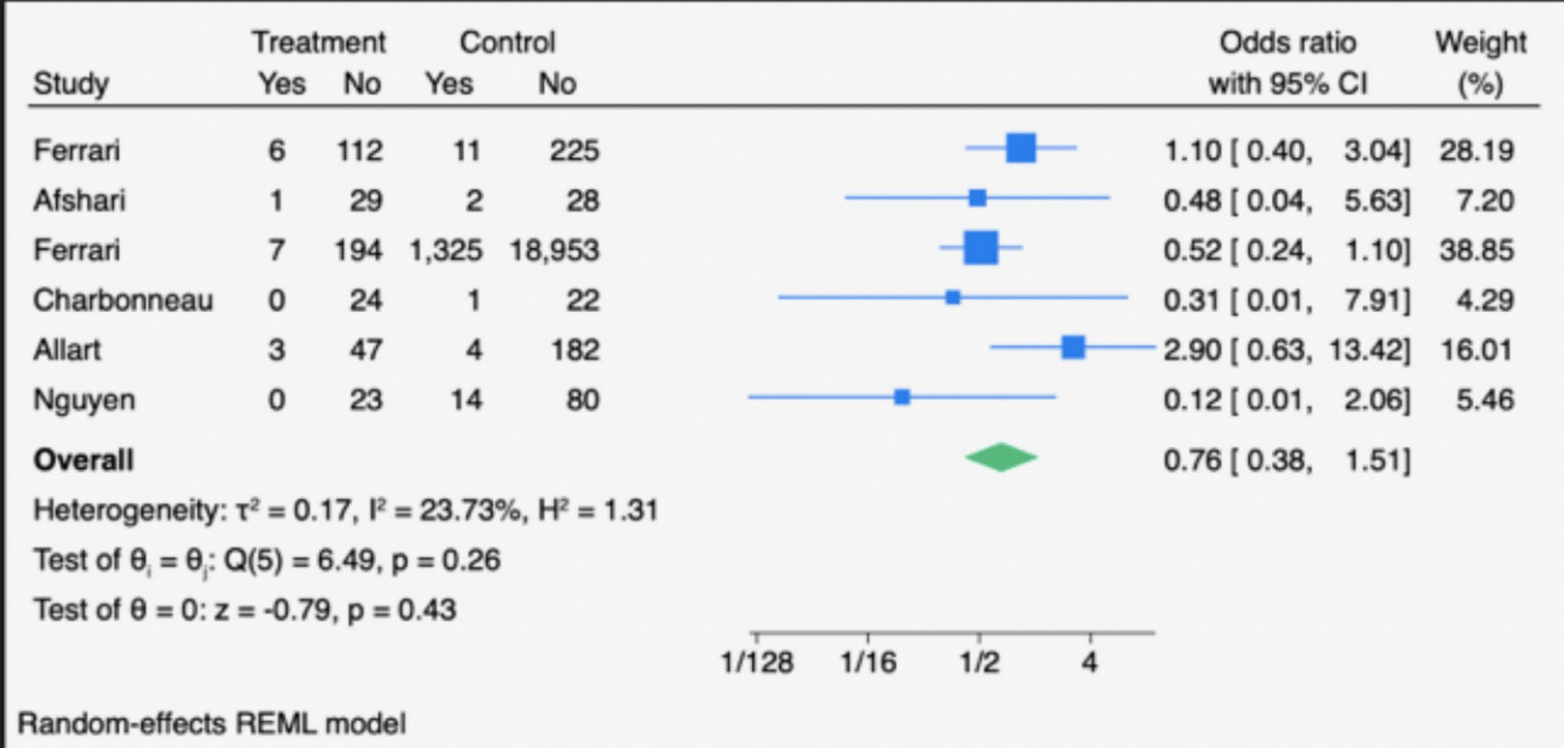
Forest plot of surgical site infection§§ following ambulatory or short-stay versus standard stoma reversal

Eight studies contributed data on overall complications, six of which used the Clavien-Dindo classification. Heterogeneity was moderate (*I*^2^ = 57.3%, *p* = 0.01), indicating variation in the magnitude but not the direction of effect. Our study showed that early discharge patients following stoma reversal experienced a statistically significant lower rate of overall postoperative complications (pooled OR 0.70, 95% CI 0.50–0.98. Six studies reported postoperative morbidity using the Clavien-Dindo classification, allowing comparison of ambulatory or short-stay pathways with standard inpatient care. Across all cohorts (*n* = 23,960), ambulatory or short-stay stoma closure (same-day or ≤ 23-h stay) involved 2611 patients, of whom 201 (7.7%) experienced a postoperative complication, compared with 2617 of 21,349 (12.3%) in conventional inpatient pathways. Minor (Clavien-Dindo I–II) events occurred in 5.6% vs 9.6%, while major (Clavien-Dindo III–IV) complications were similar at 1.9% vs 2.2%, and mortality remained low in both groups (0.2% vs 0.5%) [21, 27, 29, 30, 32–34].

A cumulative synthesis of the remaining early discharge literature, which reports outcomes using composite morbidity and standard postoperative endpoints rather than formal Clavien-Dindo grading, similarly suggests that ambulatory or short-stay stoma closure does not confer an excess risk of overall complications, readmission, reoperation, or short-term mortality compared with conventional inpatient pathways when applied in carefully selected patients [6, 9, 28, 31, 35, 36].

### Sensitivity analysis

For overall complications, the effect estimate was preserved and strengthened (pooled OR 0.55, 95% CI 0.37–0.83) with complete resolution of between-study heterogeneity (I^2^ = 0%). Non-significant effects persisted for postoperative ileus (OR 0.40, 95% CI 0.10–1.57), surgical site infection (OR 0.90, 95% CI 0.33–2.42), anastomotic leak (OR 1.32, 95% CI 0.23–7.58), and readmission (OR 0.91, 95% CI 0.37–2.26). These findings suggest that the observed reduction in overall complications is not driven by registry-level confounding, although small event counts in the remaining studies preclude firm conclusions for individual outcomes. Full sensitivity forest plots are provided in the supplementary materials*.*

## Discussion

### Main findings

Our study showed that early discharge patients following stoma reversal experienced a statistically significant lower rate of overall postoperative complications (pooled OR 0.7, 95% CI 0.5–0.98) compared with standard inpatient admission pathways. However, this difference should not be interpreted as a direct therapeutic effect of same-day surgery itself. Rather it most likely reflects the fact that clinicians appropriately selected patients at lower baseline risk of complications, typically patients with lower ASA grades and BMI, without significant comorbidities, while higher-risk patients continued to be managed through conventional admission pathways. Consistent with this, the three complications that most commonly mandate prolonged inpatient observation (anastomotic leak, postoperative ileus, and surgical site infection) occurred at similar rates between early discharge and standard cohorts, and there was also no statistically significant difference in unplanned return to hospital at 30 days (pooled OR 0.97, 95% CI 0.78–1.19). Taken together, these findings suggest that the lower overall complication rate observed in early discharge pathways is driven primarily by rigorous patient selection, augmented by structured perioperative care bundles, including clear discharge criteria, and protocolised follow-up, rather than mitigation of any single feared complication.

### Context within existing literature

Since 2003, uptake of early discharge following reversal has been slow, with Ferrari et al. showing that same-day discharges made up only 4.1% of reversals 13 years later in 2016 [32]. This slow progress likely stems from concerns regarding readmission rates and postoperative complications.

The findings of this review are concordant with those of Archer et al. [39], whose review and meta-analysis included 24,628 patients comparing short-stay (≤ 24 h) and long-stay (> 24 h) admissions for loop ileostomy reversal. They found moderate certainty evidence of no difference in readmission rates between groups (RR 0.98, 95% CI 0.75–1.28), and found that short stays may reduce non-reoperative complications (RR 0.44, 95% CI 0.31–0.62, *p* < 0.01). This mirrors the direction and magnitude of effect seen in the present analysis and collectively provides a growing evidence base supporting the safety of early discharge.

More recently, Ferrari et al. (2024) compared 125 same-day discharge (SDD) patients with 250 matched controls managed through a standard enhanced recovery pathway (ERP) and found a significantly lower 30-day complication rate in the SDD group (14.8% vs. 25.7%, *p* = 0.025), driven primarily by a lower incidence of postoperative ileus (9.6% vs. 14.8%, *p* = 0.034), with no significant differences in readmission (9.6% vs. 9.2%) or reoperation rates [32]. These findings reinforce the observation from the present meta-analysis that early discharge pathways do not appear to increase the risk of readmission while conferring a reduction in overall morbidity.

### Addressing key clinical concerns

The reluctance to adopt early discharge pathways likely centres on three principal clinical concerns: anastomotic leak, postoperative ileus, and surgical site infection. Regarding anastomotic leak, the present meta-analysis suggested no significant difference in leak rates between early discharge and standard admission cohorts (pooled OR 1.31, 95% CI 0.24–7.32), albeit without reaching statistical significance. Importantly, anastomotic leak following loop ileostomy reversal typically presents several days postoperatively rather than in the immediate postoperative period, and its low overall incidence (< 1% in most series) supports the safety of early discharge provided patients have access to rapid reassessment pathways. Emerging models of remote postoperative monitoring, including mobile application–based follow-up as described by Lee et al. [8], and scheduled community nursing visits, offer a mechanism for early detection of complications without mandating prolonged inpatient observation.

Postoperative ileus remains a more nuanced concern. The pooled analysis favoured the early discharge cohort (OR 0.49, 95% CI 0.16–1.55), although this did not reach statistical significance. Several factors may contribute to ileus risk in this population.

A major modifiable contributor is perioperative opioid exposure, which directly inhibits gastrointestinal motility through μ-opioid receptor activation and is consistently identified as an independent risk factor for postoperative ileus in colorectal surgery [40, 41]. Minimisation of opioid analgesia through multimodal approach to analgesia, including use of non-opioid analgesia, regional blocks, and non-steroidal anti-inflammatories, is a cornerstone of early discharge pathways [42, 43]. It is likely that patients discharged earlier in our study avoided protracted inpatient opioid administration which may have itself contributed to lower observed ileus rates in the early discharge cohort.

Given similar readmission rates alongside a numerically lower pooled ileus rate in the early discharge cohort, two explanations warrant consideration. First, early discharge pathways, through opioid-sparing analgesia, early enteral intake, and early mobilisation, may genuinely reduce ileus incidence. Second, milder episode of ileus may be managed in the community via structured telephone follow-up, outpatient review, or day-assessment units, without triggering formal readmission. Only a minority of studies described such community pathways and prospective studies with standardised outpatient outcome reporting are required to distinguish these possibilities [6, 9, 27, 29, 32].

Prolonged time with a diverting stoma is associated with atrophy of the defunctioned distal limb, which may predispose to functional obstruction upon restoration of continuity. Turner et al. [44] demonstrated that postoperative complications at the time of reversal increased progressively from 8% when reversed within 90 days to 54% when reversed beyond 720 days, and more recent data from Cai et al. (2024) [38, 45] confirmed previous assumptions that delayed reversal, beyond 12 months in their case, is associated with higher rates of postoperative ileus and delayed recovery of gastrointestinal function [46]. Additionally, extensive adhesiolysis required during delayed reversal may independently increase ileus risk [47, 48]. The early discharge pathway, by selecting patients earlier in their postoperative course and employing ERAS-driven perioperative optimisation, may mitigate some of these contributing factors [4, 5].

Surgical site infection rates were comparable between groups (pooled OR 0.76, 95% CI 0.38–1.51) with low heterogeneity (*I*^2^ = 23.7%). The risk of SSI following stoma closure is strongly influenced by the method of skin closure. A recent meta-analysis of eight randomised controlled trials by Rondelli et al. (2025) [49] demonstrated that circular purse-string closure (CSC) was associated with a markedly lower SSI rate compared to primary linear suture (4% vs. 27%; OR 0.11, 95% CI 0.06–0.21; *p* < 0.00001, *I*^2^ = 0%), with no difference in operative time or incisional hernia rate. Similarly, Carannante et al. (2025) confirmed in a meta-analysis restricted to high-quality studies that purse-string closure significantly reduces SSI after stoma reversal [50]. Adoption of purse-string closure as part of an early discharge protocol may further reduce SSI risk in this cohort, though the technique was not consistently reported across the studies included in the present review.

### Mechanism of benefit

The reduction in total complications without a statistically significant reduction in any single specific complication warrants consideration. Several factors may underlie this pattern.

Firstly, the early discharge pathway mandates rigorous perioperative optimisation, including careful patient selection (typically younger patients with lower ASA grades and lower BMI), protocol-driven intraoperative techniques including opiate-sparing analgesia, and structured postoperative assessments against explicit discharge criteria [51, 52]. This bundled approach aligns with the principles of Enhanced Recovery After Surgery (ERAS), which have been shown to reduce postoperative complications and length of stay across colorectal surgery broadly [53, 54]. The extension of ERAS principles specifically to stoma reversal has been advocated, though this remains an emerging area of practice.

Secondly, prolonged hospitalisation itself introduces risks, including nosocomial infection, thromboembolic events, hospital-acquired deconditioning, and delayed return to functional baseline. Expedited discharge may mitigate these hospital-acquired risks, which could contribute to the overall reduction in complications observed [55].

Looking ahead, there is a clear need for prospective, multicentre randomised trials that apply standardised definitions for anastomotic leak (ISREC), postoperative ileus (Vather or I-FEED criteria), and overall complications (Clavien-Dindo), to reduce heterogeneity and allow more robust comparisons between early discharge and standard pathways. Such work should be adequately powered for low-frequency but high-impact events, such as anastomotic leak, and integrate patient-reported outcomes, quality-of-life measures, and cost-effectiveness analyses to capture the broader impact of early discharge beyond traditional surgical endpoints.

### Patient selection and discharge criteria

The present analysis highlights the importance of patient selection. The cohort studied was relatively young (mean age 57.6 years) and fit (majority ASA Grade II), and predominantly underwent loop ileostomy closure in the context of prior malignancy surgery. Discharge criteria varied considerably across included studies; five studies specified explicit criteria including tolerance of oral diet, independent ambulation, and in some cases, return of bowel and bladder function [9, 27, 29, 32, 35], while the remainder either did not specify criteria or left discharge to the discretion of the operating surgeon. This heterogeneity in discharge criteria likely contributes to the moderate to substantial heterogeneity observed in the pooled estimates for postoperative ileus (*I*^2^ = 65.4%) and overall complications (*I*^2^ = 57.3%).

An important consideration not captured in the included studies is the impact of anastomotic height on postoperative bowel function. Patients who have undergone ultralow anterior resection with loop ileostomy may experience significant bowel dysfunction following reversal, including faecal urgency, incontinence, and clustering of stools, collectively termed low anterior resection syndrome (LARS). LARS has been reported in up to 64% of patients following sphincter-preserving rectal surgery, with lower anastomotic height identified as an independent risk factor for major LARS [55–58]. These patients may require prolonged inpatient observation to manage symptoms and to exclude *Clostridioides difficile* infection (CDI), which has been reported at an incidence of 3.5–4% following loop ileostomy reversal, approximately fourfold higher than after other elective colorectal procedures [59, 60]. In contrast, patients with higher rectal or sigmoid anastomoses are less likely to experience severe bowel dysfunction and may be more suitable candidates for same-day discharge. None of the studies included in this review stratified outcomes by anastomotic height, representing a significant gap in the current evidence base. Identifying robust predictors of successful same-day discharge and embedding them into simple, validated risk tools would help standardise patient selection and could support wider but safe dissemination of early discharge stoma reversal protocols.

The importance of appropriate patient selection is further underscored by the recent experience of Wang et al. (2025) [37], who reported outcomes of ileostomy closure performed at centres offering early discharge pathways. They found higher 30-day readmission rates in the ambulatory surgery centre cohort compared to a hospital-based cohort (42% vs. 13%, *p* = 0.027), potentially reflecting the logistical limitations of the ambulatory surgery centre setting including the lack of immediate access to inpatient observation and resources for managing complications. This suggests that while early discharge is safe, the infrastructure supporting it remains critical. This includes on-call access to surgical teams, clear patient education regarding warning signs, and rapid pathways for reassessment in the event of postoperative complications.

Furthermore, in the absence of adequately powered randomised data, a cautious risk-stratified approach to early discharge following reversal is justified at present. Current evidence may reflect potential benefit in ASA I/II patients under 70 years of age, with minimal cardiorespiratory comorbidities and with adequate social support, living in close proximity to the treating hospital. However, extending early discharge pathways to ASA > III, frail or socially isolated patients cannot currently be recommended and should be restricted to the setting of prospective trials, where these concerns can be better accounted for.

Lastly, standardised, prospectively validated discharge criteria incorporating objective thresholds for analgesic requirement, oral intake, ambulation, and passage of flatus are urgently needed. In their absence, early discharge pathways risk being applied inconsistently, limiting meaningful comparison of outcomes between centres. A consensus-derived discharge checklist would be a valuable next step.

### Limitations

Several limitations warrant acknowledgement. Firstly, most included studies were retrospective, introducing selection and confounding bias; patients chosen for early discharge were typically younger, fitter, and at lower operative risk, which may overestimate benefit in unselected populations. Secondly, definitions of anastomotic leak, ileus, and SSI were heterogeneous and largely author-defined, with no study adopting ISREC or Vather/I-FEED criteria and inconsistent SSI classifications, limiting comparability and contributing to statistical heterogeneity. Third, relatively small event numbers for individual complications, particularly anastomotic leak, restricted statistical power to detect clinically meaningful differences. With low events and wide confidence intervals, our results can therefore only be interpreted as absence of evidence of harm, rather than evidence of absence, and more robust datasets are needed for future studies to reach statistical significance.

Additionally, only one randomised controlled trial was available, reducing the overall certainty of the evidence base. Furthermore, reporting of anastomotic technique and skin closure was inconsistent, with fewer than one-third of studies providing extractable data. Although large registry cohorts dominate the pooled sample, and may plausibly dilute technique-specific effects, this assumption is untested. Given the magnitude of effect demonstrated for purse-string closure on SSI in recent meta-analyses, under-reporting of these technical variables represents a critical weakness in the available evidence base and a priority for standardised reporting in future trials.

Postoperative ileus was inconsistently reported among studies most commonly captured when it precipitated readmission or emergency presentation, or at routine 30-day clinic follow-up. No study adopted consensus definitions and subclinical or self-limiting ileus managed in the community is therefore likely under-reported in the early discharge cohort, and pooled estimates for POI should be interpreted with this detection bias in mind. Finally, there is no universally agreed definition of “same-day discharge” in this context; some studies evaluated 23-h stay protocols, others reported calendar-day discharge, and several did not clearly distinguish between true day-case and < 24-h stays, meaning our intervention group spans both same-day and short-stay pathways and likely contributes to heterogeneity in the pooled estimates [61]. In addition, only six of the twelve included studies graded complications using the Clavien-Dindo system, whereas the remainder relied on non-standardised or descriptive reporting, further limiting direct comparability of complication severity across studies and constraining pooled analyses of complication grade.

## Conclusion

In this systematic review and meta-analysis, early discharge following stoma reversal was associated with a modest but statistically significant reduction in overall postoperative complications, without a clear effect on individual complications or readmission. This is most likely reflective of patient selection in these cohorts rather than an inherent benefit of early discharge following reversal. These findings suggest that same-day discharge of loop ileostomy patients may confer benefit as part of a comprehensive perioperative care bundle, although higher-quality trials are required to define its role more precisely.

## Supplementary Information

Below is the link to the electronic supplementary material.

ESM 1 (19.2 KB DOCX)

## Data Availability

Not applicable; No original, individual patient data was collected or generated. All data generated or analysed during this study are included in this published article and its supplementary materials.

## Declarations

### Permission to reproduce material from other sources

Not applicable; no copyrighted material, figures, or tables used. No materials requiring permission for reproduction used.
