# Supplementary material for: Safety and feasibility of early discharge (≤ 23 h) following loop ileostomy reversal: a systematic review and meta-analysis
Source: Int J Colorectal Dis. 2026 May 30;41(1):126. doi: 10.1007/s00384-026-05150-6 (PMC13433468; doi:10.1007/s00384-026-05150-6)
Supplement: Supplementary file 1 [file 384_2026_5150_MOESM1_ESM.docx]

Supplementary Material:

1. Full search string:

Databases: *PubMed, Scopus, Embase*

Language: English

From database inception – 16 December 2025

Search:[tiab] ("Loop" OR "defunctioning" AND "ileostomy" OR "colostomy")

AND ("reversal" OR "restoration")

AND ("ambulatory"

OR "same-day" OR "outpatient" OR "discharge" OR "complication"

OR "follow-up" OR "inpatient")

2. GRADE Summary of Findings Table

| **Outcome** | **Studies (n)** | **Patients (n)** | **Pooled OR (95% CI)** | **Heterogeneity (I²)** | **Risk of Bias** | **Inconsistency** | **Indirectness** | **Imprecision** | **Publication Bias** | **Certainty (GRADE)** |
| --- | --- | --- | --- | --- | --- | --- | --- | --- | --- | --- |
| **PRIMARY OUTCOMES** | | | | | | | | | | |
| **Anastomotic Leak** | 4 | 515 (Afshari, Allart, Nguyen, Wang) | OR 1.31 (0.24 – 7.32) | I² = 33.2% p = 0.03 | Serious (-1)¹ | Not serious | Serious (-1)² | Very serious (-2)³ | Undetected (-1)⁴ | ⊕◯◯◯ Very Low |
| **Postoperative Ileus** | 5 | 23,936 (Charbonneau, Allart, Ferrari 2025, Taylor, Wang) | OR 0.49 (0.16 – 1.55) | I² = 65.4% p = 0.03 | Serious (-1)¹ | Serious (-1)⁵ | Serious (-1)² | Very serious (-2)⁶ | Undetected (-1)⁴ | ⊕◯◯◯ Very Low |
| **Surgical Site Infection (SSI)** | 6 | 22,836 (Ferrari 2024, Charbonneau, Afshari, Allart, Ferrari 2025, Nguyen) | OR 0.76 (0.38 – 1.51) | I² = 23.7% p = 0.26 | Serious (-1)¹ | Not serious | Serious (-1)² | Serious (-1)⁷ | Undetected (-1)⁴ | ⊕⊕◯◯ Low |
| **SECONDARY OUTCOMES** | | | | | | | | | | |
| **Unplanned 30-day Readmission** | 9 | 8,040 (Bhalla, Charbonneau, Afshari, Allart, Azin, Taylor, Nguyen, Wang, Ferrari 2024) | OR 0.97 (0.78 – 1.19) | p = 0.11 (I² n/r) | Serious (-1)¹ | Not serious | Serious (-1)² | Not serious | Undetected (-1)⁴ | ⊕⊕◯◯ Low |
| **Overall Postoperative Complications** | 8 | 23,960 (CD-graded cohort; 6 studies with CD, 2 without) | OR 0.70 (0.50 – 0.98) | I² = 57.3% p = 0.01 | Serious (-1)¹ | Serious (-1)⁵ | Serious (-1)² | Not serious | Undetected (-1)⁴ | ⊕⊕◯◯ Low |

3. Footnotes & GRADE Rationale

¹ Risk of bias: Predominantly retrospective observational design; systematic selection of fitter, younger patients for ambulatory discharge introduces selection and confounding bias.

² Indirectness: Outcome definitions were largely author-defined. No study applied ISREC criteria (anastomotic leak), Vather/I-FEED criteria (ileus), or standardised CDC criteria (SSI). "Ambulatory" encompasses both true same-day and ≤23-hour stays.

³ Very serious imprecision (anastomotic leak): Wide CI spanning large harm and benefit (OR 0.24 – 7.32); very low event count; CI crosses the null well beyond any minimal important difference.

⁴ Publication bias: Fewer than 10 studies per outcome precluded funnel plot assessment. Selective reporting favouring positive ambulatory programmes is plausible.

⁵ Serious inconsistency: I² = 65.4% (ileus) and I² = 57.3% (overall complications) exceed the 50% threshold. Likely driven by variation in discharge timing definitions, ERAS rigour, and patient selection.

⁶ Very serious imprecision (ileus): Wide 95% CI (0.16 – 1.55) crosses the null; small event counts in several contributing studies.

⁷ Serious imprecision (SSI): 95% CI (0.38 – 1.51) crosses the null. Low I² but limited absolute event count in ambulatory cohort.

GRADE certainty levels: ⊕⊕⊕⊕ High | ⊕⊕⊕◯ Moderate | ⊕⊕◯◯ Low | ⊕◯◯◯ Very Low

All observational studies start at Low certainty. One RCT (Charbonneau et al. 2024, n=47) was rated low risk of bias (Cochrane RoB-2) but insufficient alone to upgrade certainty.

PROSPERO: CRD420251252408
